# Visual discrimination down to the analytical limit of detection in colorimetric lateral flow biosensors *via* streamlined image processing

**DOI:** 10.1039/d6ra08921f

**Published:** 2026-11-10

**Authors:** Natalia-Maria Christopoulou, Panagiotis K. Tsikas, Theodore K. Christopoulos

**Affiliations:** a Analytical/Bioanalytical & Nanotechnology Research Group, Department of Chemistry, University of Patras Rio Patras 26504 Greece christopoulou.n@ac.upatras.gr panostsikas@upatras.gr

## Abstract

For point-of-care diagnostics, the visual limit of detection (LOD) is a critical parameter directly affecting diagnostic accuracy, as clinical decisions rely heavily on naked-eye classification. Improving visual detection while maintaining operational simplicity is highly advantageous for clinical testing. Here, colorimetric lateral flow biosensors for SARS-CoV-2 nucleocapsid protein (N-protein) and *Streptococcus pyogenes* antigen (Strep-A) were employed as models. The effect of N-protein concentration was studied in the range of 0–300 pM. The analytical LOD, determined both by longitudinal intensity profiling with subsequent test zone peak area integration, and by fixed region-of-interest analysis, was approximately 2 pM, whereas the visual LOD was 10–15 times higher (about 20–30 pM). Similar results were obtained for Strep-A. Within this concentration gap, human visual perception fails to resolve the colorimetric signal, leading to false-negative readouts despite the analyte level exceeding the analytical LOD. We demonstrate that a streamlined workflow based on histogram equalization of sensing membrane digital images reliably resolves subvisual, true analytical signals, effectively bridging the gap between the visual and analytical LOD. By redistributing pixel intensities *via* a cumulative distribution function, this process amplifies subtle reflectance intensity differences that remain imperceptible to the human eye. Robustness studies confirm that this resolution enhancement remains effective across both uncompressed TIFF and compressed JPEG formats, with compressed blank controls (0 pM) remaining free from non-specific signals. Because it operates exclusively on digital records without modifying the physical sensor architecture, this accessible, data-validated strategy can be combined with chemical signal-amplification methods to achieve synergistic performance improvement effects.

## Abstract 2

For point-of-care diagnostics, the visual limit of detection (LOD) is a critical parameter directly affecting diagnostic accuracy, as clinical decisions rely heavily on naked-eye classification.

## Graphical abstract

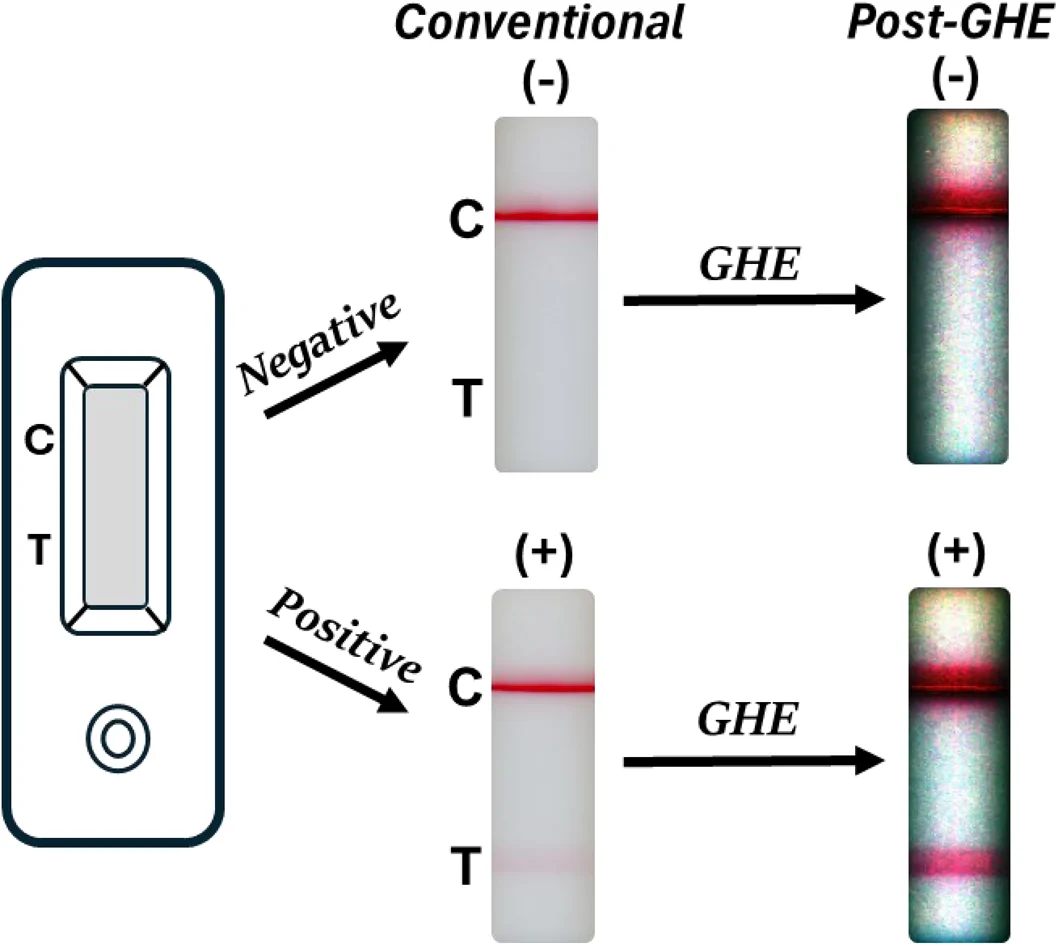

## Introduction

Lateral flow sensors are ideally suited for on-site testing, including point-of-care and field applications, across healthcare, agri-food, and environmental sectors, without the need for a laboratory infrastructure.^1–4^ Their widespread adoption as rapid- or self-diagnostic tests is primarily due to their ability to provide prompt visual readouts, enabling direct interpretation by the naked eye. Other advantages include low cost, portability, simple operation and minimal requirements for equipment and trained personnel. A lateral flow biosensor strip consists of a sample pad, a conjugate pad, a sensing membrane, and an absorbent pad. The test zone (TZ) and the control zone (CZ) are formed in the sensing membrane. Signal generation is based on the analyte-mediated accumulation of colored nanoparticles (usually gold nanoparticles) on the test zone.

Visual detection capability of lateral flow biosensors is the most critical performance parameter because it directly affects diagnostic accuracy. Low biomarker concentrations, *i.e.*, weakly positive samples, generate faint, poorly defined test zones approaching the boundaries of human optical contrast perception. The weak signals introduce severe subjectivity into the diagnostic interpretation of the test results leading to misclassification of faint true positive zones as negative results (false negatives). On the other hand, the inability to visually differentiate weak nonspecific binding from a true positive signal, at low biomarker concentrations, leads to false positive results. Thus, in several cases, the decisions determining health or disease status, such as confirming the presence of a pathogen, rely on this highly subjective visual classification. For these reasons, improving visual detectability, while keeping the simplicity and practicality of the tests, is critical for decentralized diagnostics. The visual limit of detection (LOD) in lateral flow biosensors is defined as the lowest analyte concentration generating a visible test zone that can be reliably distinguished from the blank (zero analyte concentration). Maximizing visual signals, by rendering the test zone distinctly sharper and more intense under standard ambient lighting conditions, is therefore essential to eliminate human subjectivity, prevent misdiagnoses, and achieve reliable performance by transforming ambiguous, borderline signals into unequivocal, high-contrast visual readouts.

Table S1 (SI) compiles various strategies reported for enhancing the detection limits of lateral flow biosensors. One prominent approach is to modulate the fluidics; since flow rate directly dictates the time available for molecular recognition and signal generation (*e.g.*, antigen–antibody binding), extending this interaction window can significantly improve performance. Passive architectural modifications include inserting fluid shunts or stacking pads,^5,6^ widening the conjugate and sample pads,^7^ or creating physical constrictions on the membrane *via* polymer structures,^8,9^ hydrophobic materials,^10^ water-soluble barriers,^11–15^ mechanical compression,^16^ or cotton threads.^17^ Alternatively, flow rate can be decreased by altering membrane porosity through electrospinning,^18,19^ or actively controlled using centrifugal forces^20,21^ or portable temperature-humidity regulators.^22^ Beyond fluidic adjustments, adding macromolecular crowders to the running buffer enhances detection capability by modulating the chemical microenvironment to facilitate binding kinetics.^23,24^ Also, thermal signals can be monitored using laser-induced surface plasmon heating of gold nanoparticles (AuNPs).^25^ Crucially, these protocols improve sensitivity without adding extra pipetting or washing steps.

Conversely, explicit chemical amplification methods focus on increasing the signal readout per binding event. Strategies include expanding the AuNP load through pre-assembled aggregates, sequential label addition, or nanorod carriers,^26^ enlarging capture-line bound AuNPs *via* catalytic silver reduction,^27,28^ or incorporating enzymatic cascades using horseradish peroxidase^29,30^ or biomimetic platinum coatings.^31^ Finally, two-dimensional paper networks have been engineered to automate these multi-step processes while retaining the inherent advantages of lateral flow sensors.^32^

In the present work, we utilized commercial lateral flow devices, previously optimized for the detection of SARS-CoV-2 nucleocapsid protein (N-protein) and *Streptococcus pyogenes* (Strep-A) antigen, as model platforms representing two of the most widely targeted analytes globally. For both assays, the visual LOD was found to be 10 to 15 times higher than the analytical LOD. This gap stems from the fundamental limitation of human vision in resolving minute variations in reflected light intensity against the highly reflective plain membrane matrix. Consequently, within this concentration window, naked-eye inspection fails to resolve the test zones leading to false-negative outcomes despite the analyte concentration exceeding the analytical LOD. To overcome this, we introduce a streamlined deterministic methodology based on histogram equalization of the membrane digital images to reliably resolve subvisual, authentic analytical signals. By algorithmically expanding the reflectance intensity profiles, this approach effectively bridges the gap between the visual and analytical LOD. Overall, this strategy enhances the diagnostic sensitivity and operational utility of calorimetric lateral flow sensors, and, because it operates exclusively *via* post-acquisition digital processing, it can be seamlessly combined with physical or chemical signal-amplification methods. Thus, the novelty of the work resides in the systematic quantitative characterization of histogram equalization enhancement on lateral flow strip membrane images, visual *versus* analytical LOD comparison and cross format robustness assessment (TIFF *vs.* JPEG).

## Experimental section

### Materials and equipment

The SARS-CoV-2 recombinant nucleocapsid protein (N-Protein) with N-terminal His-tag expressed in *E. Coli* (predicted Mr 47 000) was purchased from OriGene Technologies (Rockville, MD). The Verino SARS-CoV-2 antigen rapid test of Wellion was obtained from local pharmacy stores. The GIMA (Hangzhou AIITest Biotech, China) Strep-A Rapid Test Cassette (Throat Swab) was also obtained from local stores. Both kits were used according to the manufacturer's instructions. The images of the sensing membranes were obtained with the flatbed scanner Epson Perfection V600 Photo (Seiko Epson Corporation, Suwa, Japan).

### Detection of SARS-CoV-2 nucleocapsid protein (N-protein)

Solutions of various N-protein concentrations were prepared by serially diluting the protein in the extraction buffer supplied with the kit. The lateral flow assay for N-protein was performed by pipetting a 60-µL aliquot of N-protein solution to the sample-application area of the device. The results are obtained 15 min after the addition of the sample.

### Detection of group A *Streptococcus pyogenes* (Strep-A) antigen

The kit contains extraction reagents ER1 and ER2, positive control and negative control. First, ER1 was mixed with ER2 at a volume ratio of ER1/ER2 = 3/2 to prepare the final extraction reagent ER. Various dilutions of the positive control were then prepared using the ER as diluent.

For the Strep-A lateral flow immunochromatographic assay (LFIA), one volume of the sample (negative control or a dilution of the positive control) was added to ten volumes of ER and then 100 µL of the resulting solution were pipetted onto the sample-application site of the device.

### Image acquisition

Images of the lateral flow cassettes were natively captured in the sRGB color space using the Epson Perfection V600 CCD flatbed scanner at a spatial resolution of 800 DPI (pixel pitch 31.75 µm). Images were saved as uncompressed 24-bit sRGB (gamma = 2.2) TIFF files (8-bit per channel).

### Global histogram equalization

The 24-bit TIFF was opened in GIMP 2.8.22 (GNU Project), a free and open-source software. A rectangular region covering a defined part of the sensing membrane was selected and processed *via* Colors > Auto > Equalize, performing a deterministic remapping of the histogram based on the cumulative distribution function (CDF).

To isolate the primary absorption band, the rectangular region of the original image was decomposed (Colors > Components > Decompose), and the green channel was extracted to serve as the primary monochromatic image for subsequent GHE processing as above.

### Determination of the analytical limit of detection

For analytical LOD determination, the calculations were based on pixel intensity values from the green channel of the membrane images. Two methods were employed:

#### Method A: full longitudinal intensity profile followed by test zone integration

The pixel intensity values from the green channel were extracted and transferred to Excel for analysis. At each longitudinal position, the mean intensity of 34 centrally located pixels across the membrane width was calculated. The resulting mean intensity values were first inverted and then plotted against the distance (in pixels) along the membrane flow path to generate a longitudinal pixel-intensity profile. A baseline equation for the test-zone was then established by linear regression of the intensity values from a series of consecutive points before and after the TZ peak. The baseline-corrected intensity values were obtained by subtracting the baseline intensity predicted by the linear regression equation from the corresponding measured intensity at each position. Finally, the baseline-corrected area under the TZ peak was calculated by trapezoidal integration.

#### Method B: fixed-ROI mean pixel intensity measurements

For each membrane image, a fixed rectangular region of interest (ROI) was positioned over the test zone. An identical ROI was positioned in the background region above the test zone. The mean intensity of all the pixels enclosed within the ROI was obtained from the histogram tool of GIMP 2.8. The net signal was calculated by subtracting the test zone ROI intensity from the background ROI intensity.

For both methods, the analytical LOD was calculated according to the equation LOD = 3.3 *s*/*m*, where *s* is the standard deviation of the blank (zero analyte concentration) measurements and *m* is the slope obtained from a linear fit of the calibration data over the low-concentration range.

## Results and discussion

### Principle of the assay

The lateral flow chromatographic immunosensors employed in this study are based on the principle of heterogeneous, non-competitive immunoassay (sandwich type) involving two antibody-mediated recognition events of the analyte. One antibody is immobilized on the test zone of the sensing membrane, whereas the other is deposited, in dry form, onto the conjugate pad as a conjugate with gold nanoparticle (AuNPs) reporters. After applying the sample mixed with running buffer, the reagents are driven along the strip by capillary forces, causing redispersion of the gold nanoparticle – antibody conjugates and enabling the antigen (analyte)–antibody interaction as the first molecular recognition event of the analyte. The solution then reaches the test zone of the sensing membrane, where a sandwich-type immunocomplex is formed. The accumulation of colored reporters in the test zone produces a red signal, indicating the presence of the antigen. In the absence of analyte, the immunocomplex is not formed, and the functionalized AuNPs migrate along the membrane without accumulating in the TZ. The excess antibody-functionalized AuNP are captured in the control zone (CZ) of the membrane. A red signal appears in the CZ, regardless of the presence of the analyte, serving as confirmation of proper sensor function. It mainly verifies the effective re-dispersion of the functionalized AuNPs and the unobstructed flow of reagents along the membrane. Upon completion of the lateral flow assay, the cassette images were captured using a flatbed scanner.

### Determination of the visual and analytical LOD for SARS-CoV-2 nucleocapsid protein

In this study, nucleocapsid protein (N-protein) samples were prepared across a concentration range of 0–300 pM. The samples were prepared by serial dilutions of the stock N-protein solution using the kit's extraction buffer as the diluent to maintain a consistent and relevant chemical environment for the analyte. The assays were performed in triplicate for each concentration level. The images of the lateral flow cassettes were captured using a flatbed scanner. By utilizing the scanner's light-tight lid enclosure and fixed internal illumination assembly, the image acquisition process isolates the membranes from external ambient light interference ensuring colorimetric consistency and uniform pixel intensity distributions across all scanned series. Subsequently, the images were opened and positioned vertically in GIMP and then a rectangular region (ROI) of the sensing membrane, enclosing both the control and test zones, was selected and cropped (excluding the cassette housing). The same ROI dimensions were used for all membrane images, so image processing was based only on the membrane background and the signals of the two zones. Fig. 1A presents the original (conventional) images of the sensing membranes across the entire concentration range. From the series of images, it is evident that the lowest concentration of N-protein that produces a visible test zone, clearly distinguishable from the background by the naked eye, is about 30 pM.

**Fig. 1:**
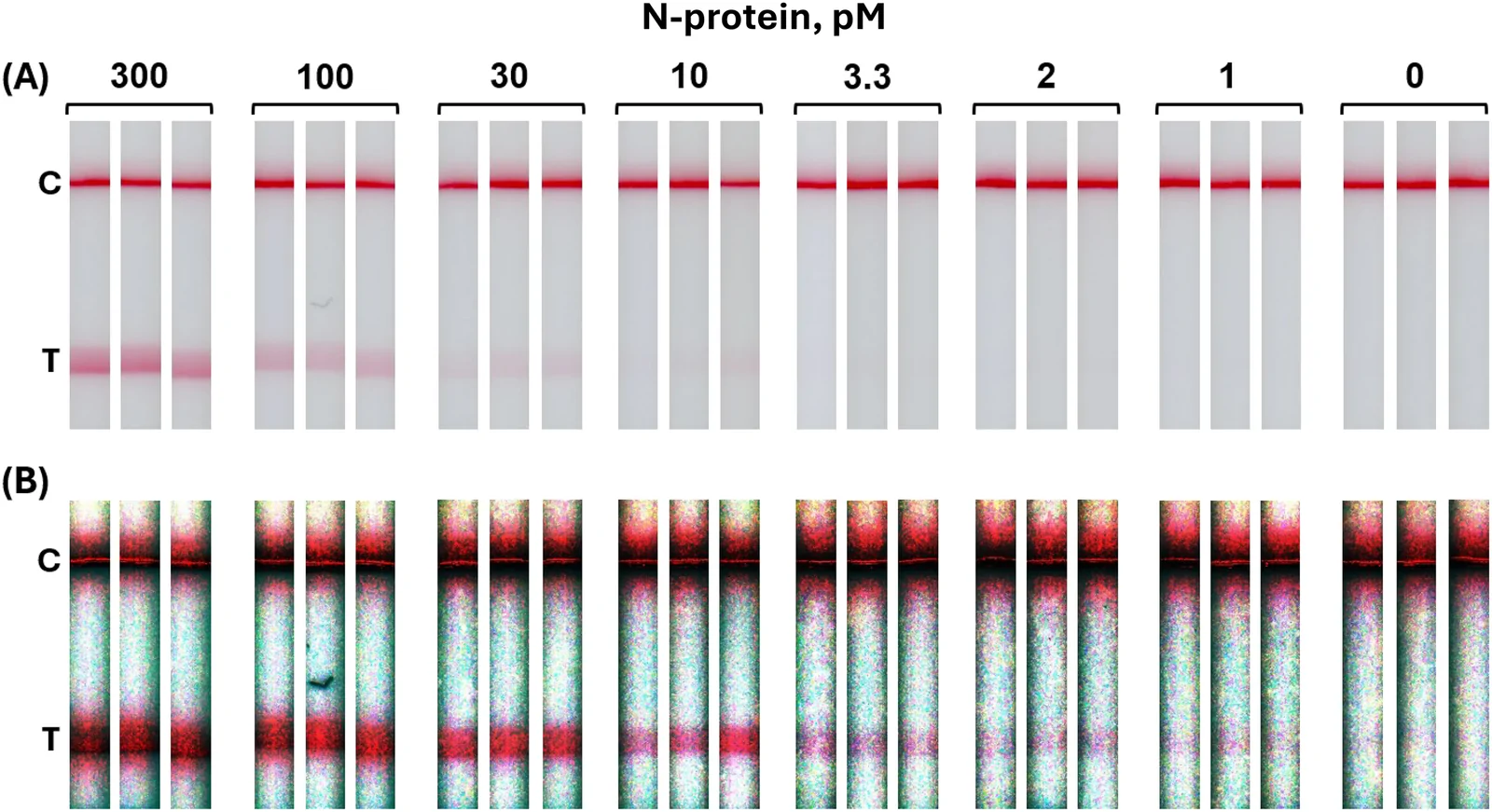
Images of the sensing membranes of lateral flow immunosensors applied to the detection of SARS-CoV-2 nucleocapsid protein (N-protein) in the concentration range of 0–300 pM. (A) Conventional images and (B) post-GHE images. All the assays were performed in triplicate. The concentrations, in pM, are shown at the top of the images. C = control zone; T = test zone.

The color images were split into their respective color channels, R, G, B and the pixel intensities of the green channel were used to create a longitudinal intensity profile for each image at each N-protein concentration level. The blank membrane (zero analyte concentration) reflects all visible wavelengths across red, green and blue channels at high intensity. The accumulated AuNPs in the test and control zones, absorb light in the green spectral region (around 520 nm) while continuing to reflect red light. Thus, the AuNP accumulation is digitally manifested by the localized drop in green reflectance rather than an increase in red reflectance. Extracting the green channel, therefore, captures the maximum reflectance intensity drop against the highly reflective membrane matrix providing higher analytical sensitivity. Specifically, at each longitudinal position, the mean intensity of 34 centrally located pixels across the membrane width was calculated. The resulting mean intensity values were first inverted and then plotted against the distance (in pixels) along the membrane flow path to generate a longitudinal pixel-intensity profile. The intensities of more than 10 000 pixels per individual image were employed for these calculations.

Fig. 2A presents the longitudinal intensity profiles for all concentration levels. Each profile in Fig. 2A, was constructed by taking the point-by-point mean value of the three longitudinal profiles obtained from the triplicate membrane images at each concentration level. The pixel intensity values are presented on a scale of 0–255. The high accumulation of AuNPs in the control zone (CZ) yields the large intensity values (up to 255) observed in the first prominent peak of the diagram. The second peak corresponds to the TZ, and its magnitude increases as a function of the N-protein concentration due to analyte-mediated accumulation of AuNPs. Fig. 2B presents a rescaled diagram of superimposed intensity profiles of the test zones focusing on the low N-protein concentrations (0, 1, 2, 3.3 and 10 pM) that are not visible to the naked eye. Even at these low levels, a distinct concentration-dependent increase of the peak height is observed. For determination of the analytical LOD by test zone peak integration (Method A), a baseline equation was first established for the TZ peak of each membrane image by linear regression of the intensity values from a series of consecutive points before and after the TZ peak. The baseline-corrected intensity values of the TZ peak were calculated by subtracting the baseline intensity predicted by the linear regression equation from the corresponding measured intensity at each position. Finally, the baseline-corrected area under the TZ peak was calculated by trapezoidal integration.

**Fig. 2:**
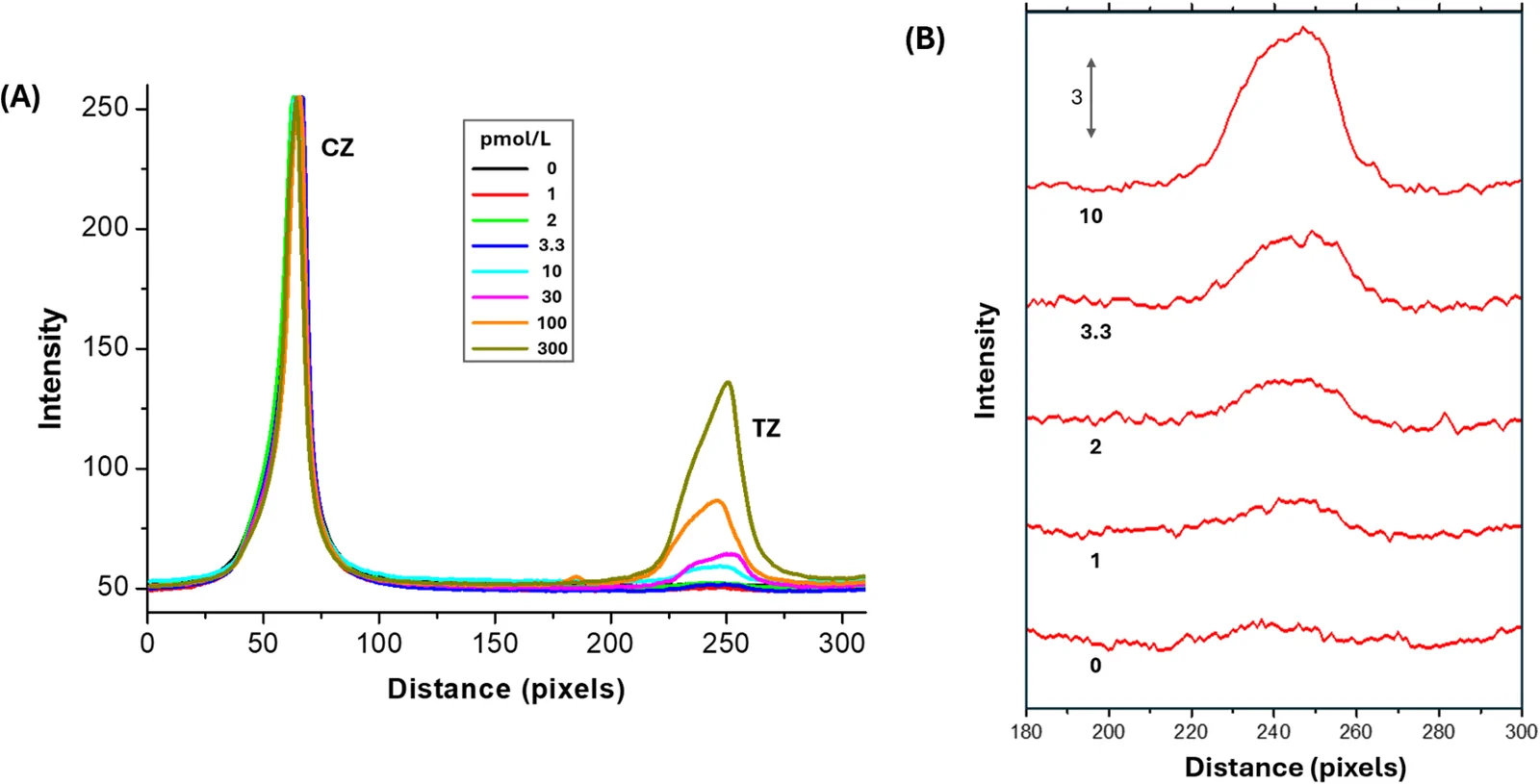
(A) Longitudinal intensity profiles of the membrane images for all N-protein concentration levels (from 0 to 300 pM) showing the intensity values as a function of the vertical distance. (B) Rescaled diagram of superimposed intensity profiles of the test zones focusing on the low N-protein concentrations (0, 1, 2, 3.3 and 10 pM) that are invisible to the naked eye. CZ = control zone; TZ = test zone.

The plot of the peak area as a function of the N-protein concentration is presented in Fig. 3A. As shown in the insert of Fig. 3A, there is a linear relationship between the peak area and the N-protein concentration in the low concentration range (0 to 10 pM). The slope (*m*) of the equation derived from the weighted least squares linear regression within this concentration range, combined with the standard deviation (*s*) of the blank peak area (zero analyte concentration), was used to calculate the analytical LOD (LOD = 3.3 *s*/*m*). According to this method (Method A), the analytical LOD was found to be 2.0 pM. Consequently, the analytical LOD is about 15 times lower than the visual LOD on which the diagnostic decisions are based. Moreover, we determined the analytical LOD by fixed-ROI mean pixel intensity measurements (Method B). A fixed-size rectangle ROI (width 40, height 25) was positioned first on the test zone and then on the background region above the test zone. The mean intensity of all the pixels enclosed within the ROI was obtained from the histogram tool of GIMP. The net signal was calculated by subtracting the test zone ROI intensity from the background ROI intensity. The background corrected ROI mean intensity graph as a function of N-protein concentration is presented in Fig. 3B. The insert of Fig. 3B shows that there is a linear relationship between the ROI mean intensity and the N-protein concentration in the low concentration range of 0 to 10 pM. The standard deviation of the mean intensity of the blank and the slope obtained from weighted least squares regression in the low-concentration range are used for the calculation of the analytical LOD. The analytical LOD determined by Method B was 1.9 pM.

**Fig. 3:**
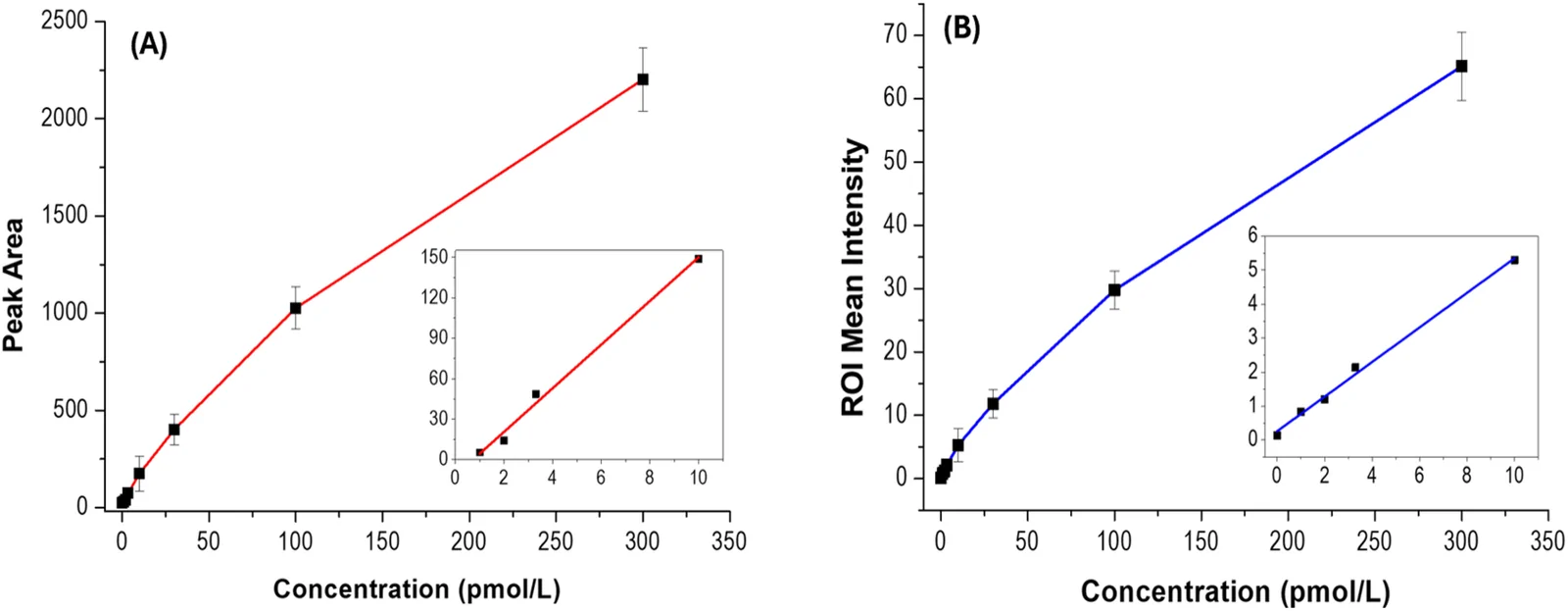
(A) Graphs showing the area under the peak of the test zone as a function of the N-protein concentration. (B) Graphs showing the ROI mean intensity as a function of the N-protein concentration.

### Resolution of subvisual analytical signals – visual discrimination down to the analytical limit of detection

Fig. 4 illustrates the effect of histogram equalization on the membrane images of lateral flow biosensors. Results for a negative sample and a low-concentration positive sample, for both SARS-CoV-2 and *Streptococcus pyogenes* (Strep-A) antigens, are presented. In the conventional (original) images of the sensing membranes, the test zones of the negative samples show absence of visual signal, while the positive samples exhibit only a faint visual signal due to the selected antigen concentrations being near the sensors' visual detection limits. After global histogram equalization (post-GHE images) of the same membranes, the test zones of the positive samples appear profoundly stronger than in the conventional images, while the negative samples remain clear of any detectable signal.

**Fig. 4:**
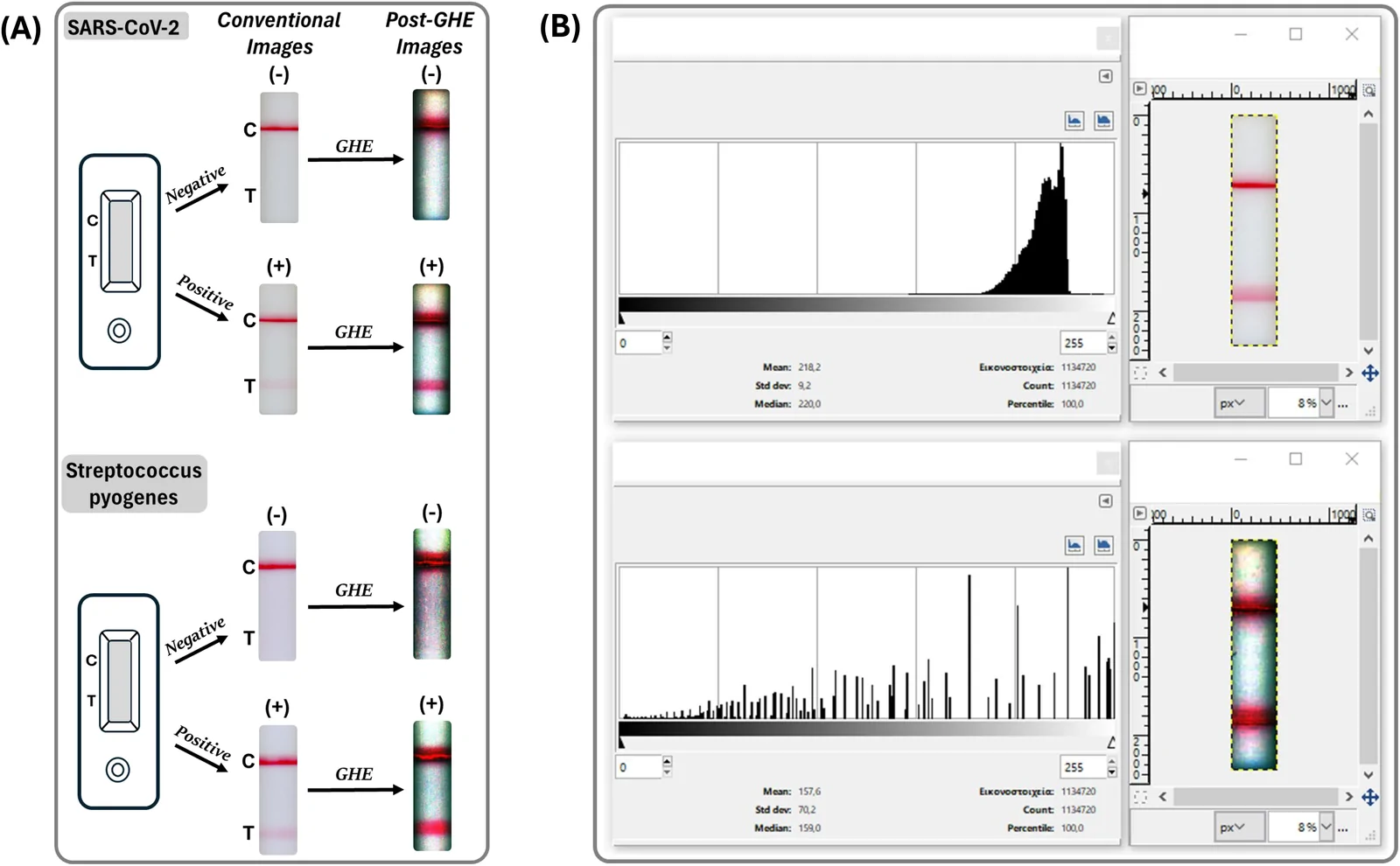
(A) Lateral flow immunochromatographic assays (LFIA) for SARS-CoV-2 and *Streptococcus pyogenes* target antigens. For each target, a negative sample (absence of antigen) and a low-positive sample (low antigen concentration) are presented. For each sample, the raw image of the sensing membrane (conventional image) and the corresponding image obtained by global histogram equalization (post-GHE image) are shown. T = test zone; C = control zone. (B) Top: conventional image and the corresponding histogram. Bottom: histogram and image after global histogram equalization.

Global histogram equalization is a deterministic technique that amplifies subvisual reflectance intensity differences otherwise obscured within the compressed histogram of a raw image. Rather than generating new data, the algorithm maximizes the analytical utility of existing information. As illustrated in Fig. 4, the brightness distribution of a conventional image of the membrane typically exhibits a narrow, highly compressed profile in the high-intensity region (200–255). This extreme statistical crowding results in a barely detectable test zone where subtle intensity variations in the high reflectance (off-white) region, though physically recorded by the sensor, remain imperceptible to the human eye. Because the human visual system cannot resolve such minute reflectance differences against a bright background, some low-intensity, genuine analytical signals are visually misclassified as negatives. GHE addresses this limitation by remapping the original pixel intensities across the full 8-bit dynamic range (0–255) *via* a cumulative distribution function (CDF). For the representative case in Fig. 4, this transformation expands the pixel intensity spread (standard deviation) from 9.2 to 70.2 and shifts the mean brightness from 218.2 to 157.6. This marked increase in dispersion confirms that the image now fully utilizes the available dynamic range, producing pronounced, resolvable transitions between the test zone and the membrane background.

Fig. 1 presents a direct comparison of the conventional and post-GHE images of the sensing membranes across the entire concentration range (0–300 pM). We observe that, across all concentration levels, the post-GHE images exhibit a substantial increase in the intensity of the test zone. In the conventional images, only the concentration levels of 30, 100 and 300 pM yield clearly visible test zones. In contrast, the post-GHE images allow the concentrations 1–2, 3.3 and 10 pM to be visually identified without ambiguity. Therefore, the post-GHE visual LOD meets the analytical LOD. Furthermore, the intensity of the test zone increases with concentration. The control zone in the processed images, as expected, is very intense. These findings indicate that the deterministic remapping of the full color-space acquisition is sufficient to resolve subvisual analytical signals and extend the visual LOD down to the analytical LOD. The direct application of GHE on the color image streamlines the analytical procedure without compromising the resolution of non-visible analytical signals. This enhances the practical utility of the method for end-users, ensuring high-sensitivity readout without the need for other complicated image processing. Fig. S1 presents the processing workflow of the images by GHE in the form of a pseudo-code (Python style) for convenience.

### Robustness of subvisual analytical signal resolution across TIFF and JPEG formats

Robustness studies were performed to investigate the efficacy of invisible analytical signal resolution across both lossless TIFF and lossy JPEG formats. Following the assay, lateral flow cassettes across the full concentration range (0–300 pM) were scanned, and the resulting acquisitions were saved simultaneously in both formats to ensure a direct comparison. Each file type was then subjected to GHE, applied either to the direct color image of the sensing membrane or the isolated green channel. As evidenced in Fig. S2, the GHE-mediated resolution of analytical signals remains highly effective across the entire concentration range for both file formats. Both formats provide visual detection down to the analytical LOD, which is 2 pM for N-protein. The only difference observed was a slight decrease in the absolute post-GHE signal intensity in the JPEG files, which is a consequence of lossy compression but does not impact the practical readout (positive *vs.* negative). Therefore, because both post-GHE images (TIFF and JPEG) reach the analytical LOD, there is no practical LOD shift to quantify across compression levels. Also, compressed negative controls consistently exhibited clean baselines post-GHE with no false-positive bands, experimentally demonstrating that standard JPEG compression does not interfere with result interpretation.

### Detection of SARS-CoV-2 in clinical samples

Nasal swab samples were self-collected by individuals who performed the test in accordance with the manufacturer's instructions (same swab for both nostrils). The swab was inserted into the extraction buffer, after which the sample was analyzed immediately or preserved at −20 °C for subsequent testing. The lateral flow assay was performed by pipetting 60 µL of the sample to the device. Fig. 5 shows images of the sensing membranes positioned side by side. In each pair, the left image depicts the conventional image, whereas the right shows the same membrane after histogram equalization.

**Fig. 5:**
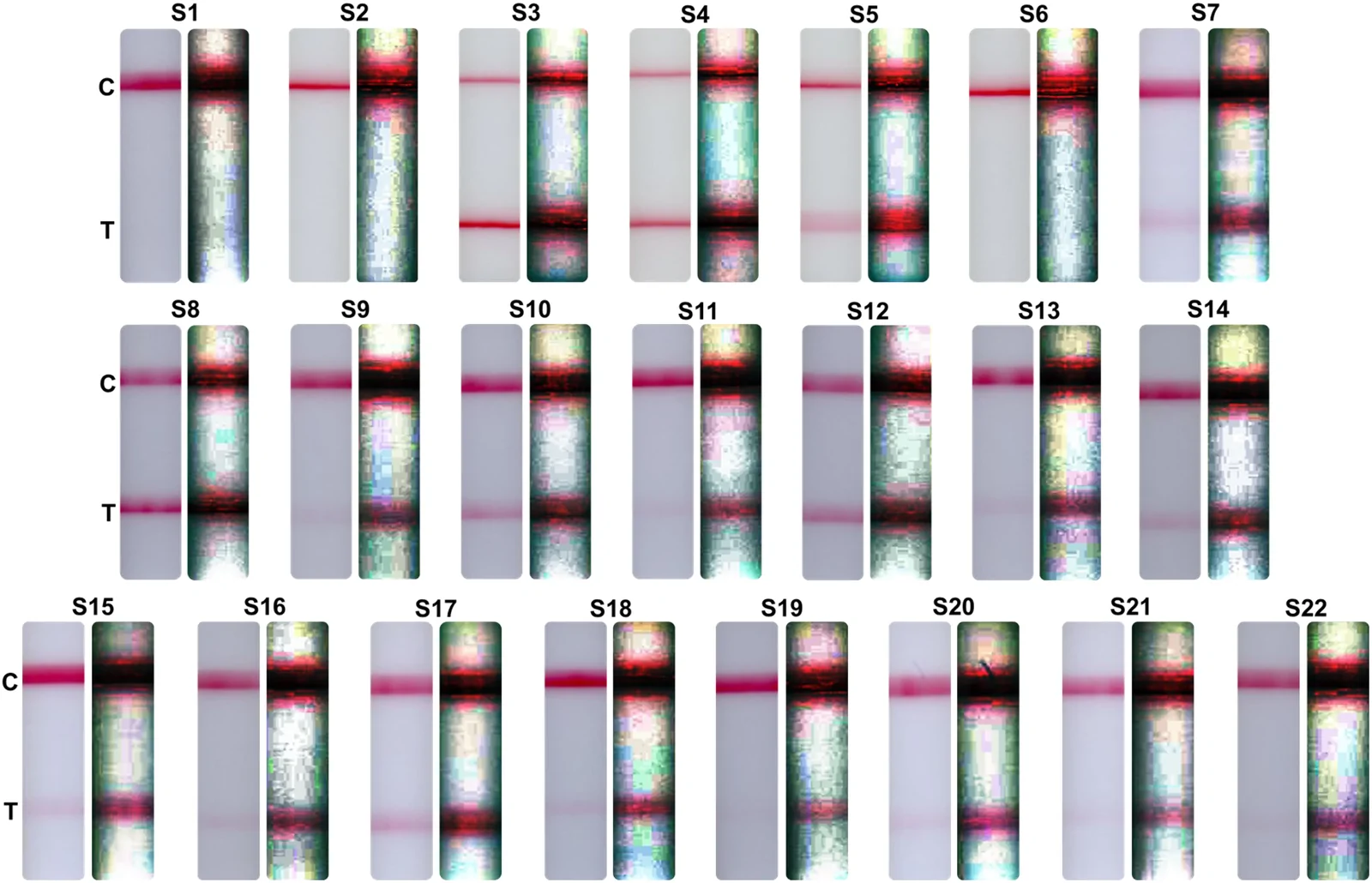
Conventional and post-GHE images of the sensing membranes of lateral flow immunosensors applied to the detection of SARS-CoV-2 in nasal swabs. The images are positioned side by side. In each pair, the left image depicts the conventional image, whereas the right shows the same membrane after global histogram equalization. C = control zone; T = test zone.

Samples S1 and S2 were negative for SARS-CoV-2, which is confirmed by both the conventional and post-GHE images. Samples S3–S6 were collected on different days from an individual who had COVID-19. Samples S4–S6 were collected one, three, and seven days after sample S3, respectively. A decrease in antigen concentration is observed in both the conventional and post-GHE images. However, the test zone in the post-GHE image remains markedly stronger. Sample S6 is negative in both the conventional and post-GHE images, reflecting the extended time elapsed since infection. Sample S7 was positive for the virus. A red line is observed at the test zone of the conventional image. The signal intensity, however, is remarkably higher after GHE. S8 was a positive sample and S9 was a sample prepared by 100 times dilution of sample S8 in the extraction buffer of the kit. We observe that the red line in the test zone of the conventional image is marginally detectable whereas after GHE, the visibility of the test zone increases dramatically providing an undoubtedly positive result. S11 and S13 were prepared by 10-fold and 20-fold dilutions of positive samples S10 and S12, respectively. The test zones of S11 and S13 are weak but are markedly enhanced after GHE. Also, low-positive samples S14–S22 show significantly improved signals after GHE. These samples were from self-tests performed some days after the disappearance of the symptoms of COVID-19. The resolution of subvisual analytical signals *via* GHE is particularly important for samples that are found to be marginally positive by the conventional image, giving a very faint test zone, whereas the test zone is clearly visible after GHE thereby facilitating the detection. Obtaining a clear and unambiguous visual result by lateral flow immunochromatographic devices is extremely desirable and important for diagnostic purposes and patient management.

### Determination of the visual and analytical LOD for Strep-A antigen

The assay was performed by mixing one volume of the sample with ten volumes of extraction reagent, after which 100 µL of the resulting solution was applied to the sample area of the device. We prepared serial dilutions of the positive control solution (included in the kit) using the extraction reagent (ER) as the diluent. The range of dilutions studied was from 100-fold to 10 000-fold. Because the concentration of the positive control was unknown, the concentrations are expressed as the reciprocal of the dilution factor multiplied by 10^4^, thereby representing relative concentration levels with respect to the positive control. The assays were performed in triplicate for each concentration level. The conventional and post-GHE images of the sensing membranes are presented in Fig. 6.

**Fig. 6:**
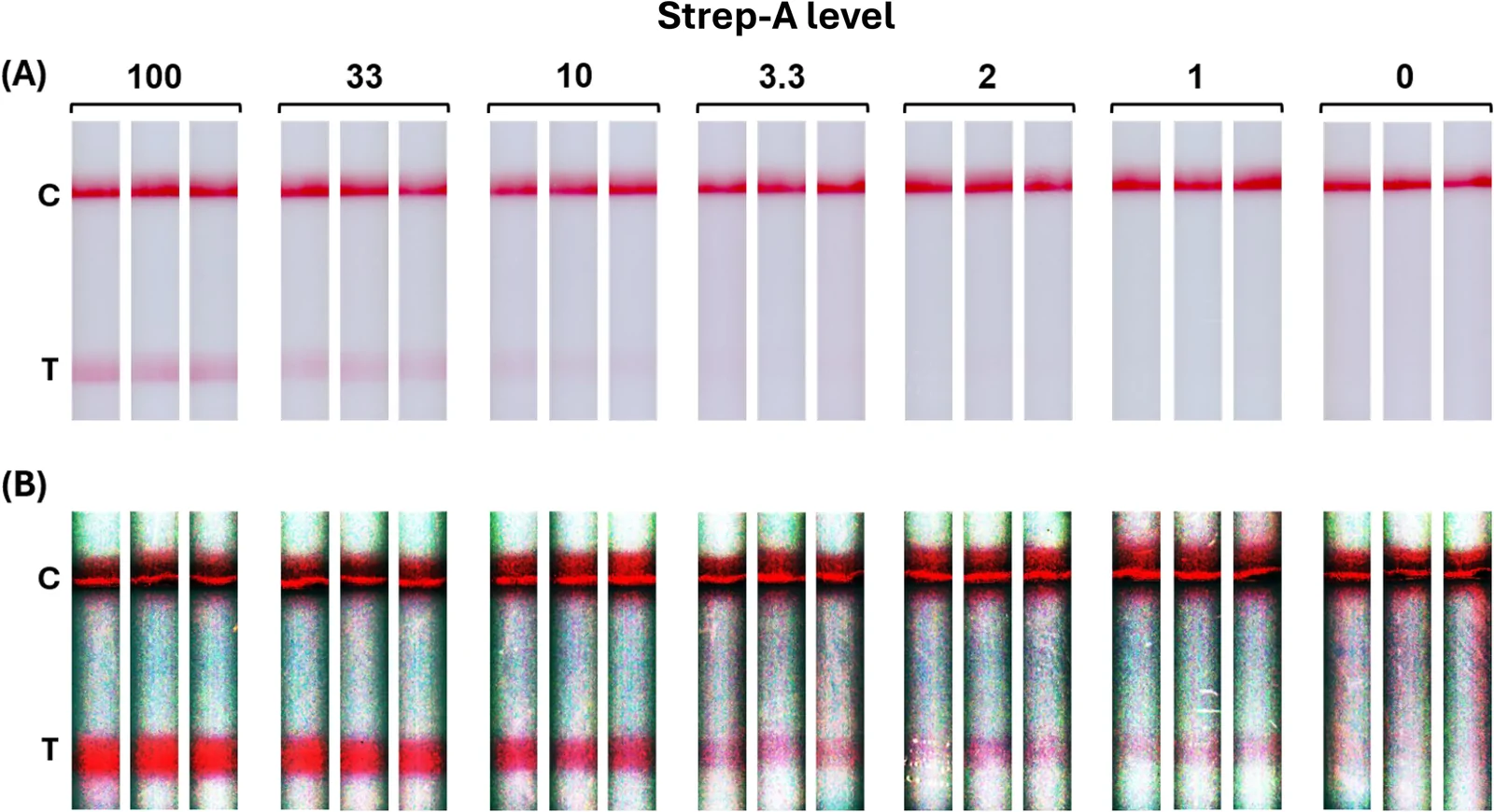
Images of the sensing membranes of lateral flow immunosensors applied to the detection of Strep-A antigen from *Streptococcus pyogenes*. (A) Conventional images and (B) post-GHE images. The range of dilutions of the positive control was from 100- to 10 000-fold. Because the concentration of the positive control was not known, the concentrations are expressed as the reciprocal of the dilution factor multiplied by 10^4^ (relative concentration levels). The relative concentrations are shown above the images. All the assays were performed in triplicate. C = control zone; T = test zone.

From the series of images, it is evident that the lowest concentration of Strep-A that produces a visible test zone, clearly distinguishable from the background by the naked eye, is between 10 and 33. Fig. 7A presents the longitudinal intensity profiles for all concentration levels. Each profile was constructed by averaging, point-by-point, the three longitudinal profiles obtained from the triplicate images at each Strep-A concentration level. Fig. 7B presents a rescaled diagram of superimposed intensity profiles of the test zones focusing on the low Strep-A concentrations (0, 1, 2, 3.3 and 10) that are invisible to the naked eye. A distinct concentration-dependent increase of the peak height is observed.

**Fig. 7:**
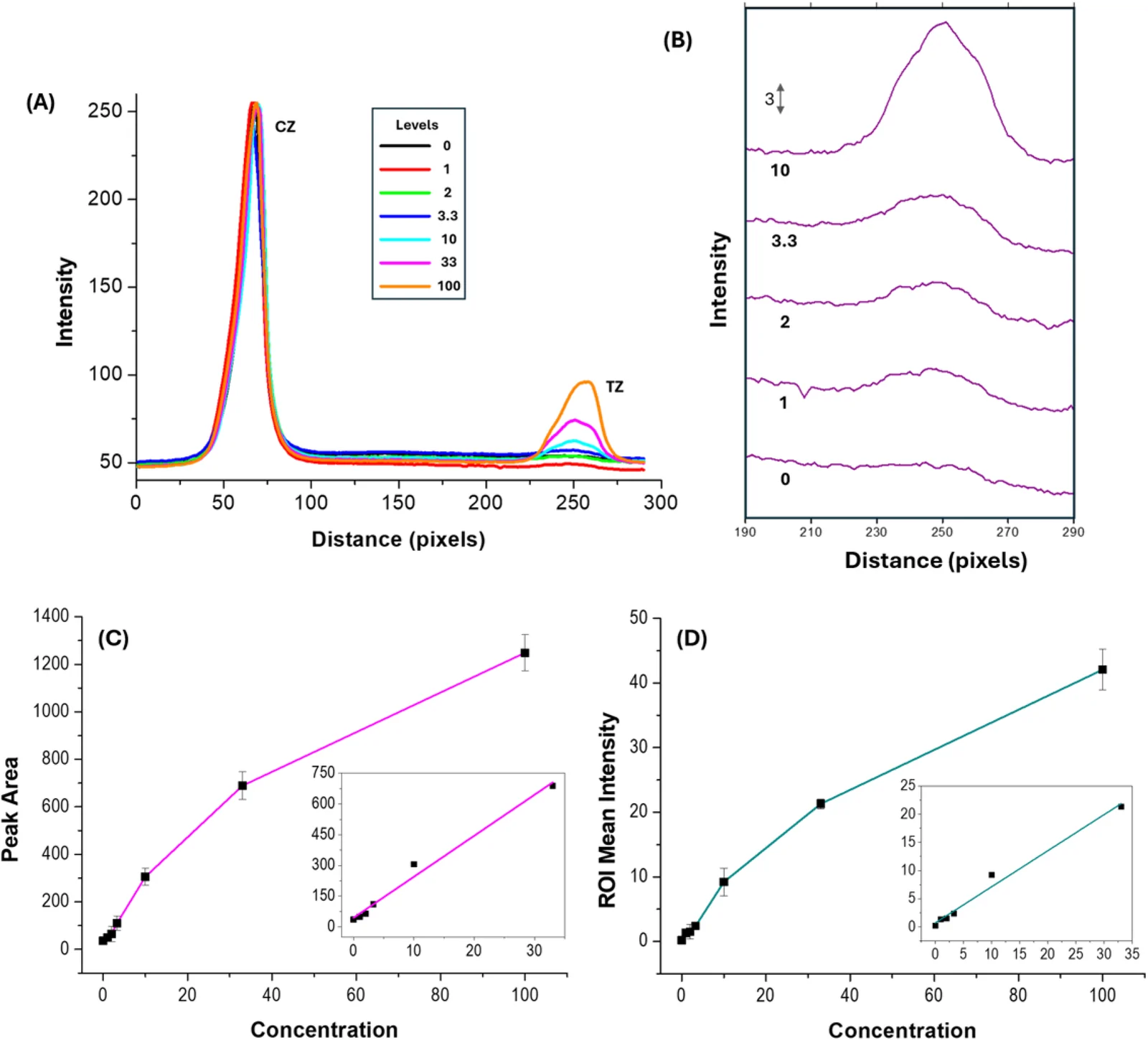
(A) Longitudinal intensity profiles of the membrane images for all Strep-A levels (from 0 to 100) showing the intensity values as a function of the vertical distance. (B) Rescaled diagram of superimposed intensity profiles of the test zones focusing on the low Strep-A levels (0, 1, 2, 3.3 and 10) that are invisible to the naked eye. (C) Graphs showing the area under the peak of the test zone as a function of the Strep-A level. (D) Graphs showing the ROI mean intensity as a function of the Strep-A level. The range of dilutions of the positive control was from 100- to 10 000-fold. Because the concentration of the positive control was not known, the concentrations are expressed as the reciprocal of the dilution factor multiplied by 10^4^ (relative concentration levels). The relative concentrations are shown in the graphs. CZ = control zone; TZ = test zone.

For analytical LOD determination by Method A, the TZ peak area was plotted as a function of Strep-A concentration (Fig. 7C). As shown in the insert of Fig. 7C, there is a linear relationship between the peak area and the N-protein concentration in the low concentration range (0 to 33). According to this method (Method A), the analytical LOD was found to be 1.7. Consequently, the analytical LOD is about 10 times lower than the visual LOD on which the diagnostic decisions are based. Moreover, we determined the analytical LOD by fixed-ROI mean pixel intensity measurements (Method B). The ROI mean intensity graph as a function of Strep-A concentration is presented in Fig. 7D. The insert of Fig. 7D shows that there is a linear relationship between the ROI mean intensity and the Strep-A concentration in the low concentration range of 0 to 33. The analytical LOD determined by Method B was 2.4. We further observe in Fig. 6, that, at all dilution levels, the post-GHE images exhibit a marked increase in the intensity of the test zone compared to conventional images, while at the zero-level, the test zone remains practically blank. The test-zone intensity increases with concentration. In the conventional images, the test zone becomes barely visible to the naked eye at a relative concentration between 10 and 33. In contrast, the post-GHE images enable visualization at much lower concentrations, namely at 1–2 and 3.3 and the color intensity increases with the concentration of Strep-A antigen. Therefore, the post-GHE visual LOD is 10 times lower than the conventional visual LOD and meets effectively the analytical LOD.

Variations in nitrocellulose membrane specifications across different commercial manufacturers, such as pore size, surface roughness and baseline material reflectivity, will naturally alter the raw image histogram. However, by dynamically stretching the pixel intensities across the full scale, GHE normalizes the plain membrane matrix and significantly sharpens the test (and control) lines. Consequently, the visual LOD improvement demonstrated in this work is highly versatile and similar performance enhancements can be expected across diverse commercial lateral flow platforms regardless of internal membrane architecture.

In the present study, all images were captured using a benchtop flatbed scanner. Smartphone-based image acquisition was not utilized. A scanner setup was selected to demonstrate the capabilities and the potential of GHE image processing under controlled, reproducible conditions. Besides providing the visual LOD improvement capabilities of GHE, the proposed platform is immediately applicable to high-throughput clinical or hospital laboratory settings. Adapting the proposed method to smartphone photography is an important next step for point-of-care decentralized deployment.^33,34^ This transition would require addressing the practical constraints inherent to smartphone-based image acquisition such as shifting ambient illumination, variable lighting angles, automatic exposure adjustments, flash and shadow. This would be achieved through software features like lighting normalization or color calibration, to separate true test-line from changing ambient light conditions.

## Conclusions

Using the colorimetric lateral flow immunochromatographic assays (LFIA) for SARS-CoV-2 nucleocapsid protein and *Streptococcus pyogenes* antigen (Strep-A) as models, we found that visual LOD is about 10–15 times higher than the analytical LOD. Within this concentration gap, the visual detection capability fails to discern the analyte even though its level is above the analytical LOD (false negative results). We have demonstrated a streamlined, deterministic framework for the resolution of subvisual analytical signals in lateral flow biosensors using histogram equalization. By redistributing pixel intensities *via* a cumulative distribution function, the image processing workflow amplifies subtle reflectance intensity differences that remain imperceptible to the human eye. The effective resolution of the subvisual, genuine analytical signals results in substantial lowering of the visual LOD down to the level of the analytical LOD. Across all runs, the blank consistently demonstrated a negative post-GHE readout confirming that noise artifacts did not cross the visual threshold. In both analytes, the post-GHE visual LOD consistently aligned with the calculated analytical LOD without yielding false positives in the blank. Robustness studies further demonstrated that the resolution of subvisual analytical signals remains effective across both lossless TIFF and lossy JPEG formats. Because it relies exclusively on open-source and public-domain tools, this strategy provides a highly accessible and cost-effective pathway for improving the visual detection capability of colorimetric point-of-care diagnostic devices. Since histogram equalization operates exclusively on the digital record of the sensing membrane without affecting the assay configuration or sensor architecture, it can be combined with chemical signal-amplification strategies to achieve a synergistic improvement effect. It is anticipated that this approach will prevent visual misclassification of colorimetric lateral flow biosensor results, and drastically minimize user error, thereby improving diagnostic accuracy at the point of care. Transferring the proposed image processing method to smartphone-based application is the logical next step for this technology, since it preserves the portability of lateral flow colorimetric biosensors and eliminates the need for expensive, bulky laboratory instrumentation in decentralized fields.

## Author contributions

Natalia-Maria Christopoulou: methodology, experimental design, resources, data extraction from images and analytical LOD calculations, formal analysis, cross-platform verification, investigation, validation, writing – review and editing. Panagiotis K. Tsikas: original observation of image enhancement by GHE, formal analysis, investigation, writing – review & editing. Theodore K. Christopoulos: writing – review & editing.

## Conflicts of interest

The authors declare no conflict of interest.

## Supplementary Material

RA-OLF-D6RA08921F-s001

## Data Availability

The data supporting this article have been included as part of the supplementary information (SI). Supplementary information is available. See DOI: https://doi.org/10.1039/d6ra08921f.
