# Supplementary material for: Visual discrimination down to the analytical limit of detection in colorimetric lateral flow biosensors via streamlined image processing
Source: RSC Adv. 2026 Nov 10. Online ahead of print. doi: 10.1039/d6ra08921f (PMC13749439; doi:10.1039/d6ra08921f)
Supplement: Supplementary file 1 [file RA-OLF-D6RA08921F-s001.pdf]

Supplementary Information (SI) for RSC Advances.
This journal is © The Royal Society of Chemistry

Supplementary Information

Visual Discrimination Down to the Analytical Limit of Detection in Colorimetric Lateral Flow Biosensors via Streamlined Image Processing

Natalia-Maria Christopoulou*, Panagiotis K. Tsikas*, and Theodore K. Christopoulos

Analytical/Bioanalytical & Nanotechnology Research Group,
Department of Chemistry, University of Patras, Rio, Patras, Greece 26504

*Corresponding authors.

Email: christopoulou.n@ac.upatras.gr; panostsikas@upatras.gr

Table S1. Strategies for improving the limit of detection of lateral flow biosensors (LOD fold improvement).

Strategy	Analyte	*LOD* fold	Ref.
Modification of strip architecture to reduce flow-rate			
Sponge shunt between CP* and M*	HepB* nucleic acid	10	5
Stacking pad	CRP* and Protein A	5	6
Increase width of CP and SP*	hIgG*	8	7
Spatial constrictions within M	Procalcitonin	10	9
	CRP	30	8
Wax pillars before TZ*	hIgG	3	10
PVA* dam before TZ	hIgG	10	11
Hydrogel before TZ	hIgG	3.3	12
Compressed barriers before TZ	SARS-CoV-2 nucleic acid	4	16
Wax barriers after TZ	hIgG	2.4	13
Trimethylsilyl barrier after TZ	SARS-CoV-2	5	14
Sugar barriers, before and after TZ	SARS-CoV-2 nucleic acid	5	15
Cotton threads between CP and M	HIV nucleic acid	4	17
Nitrocellulose fibers on M	hCG, LH*	50	18
Poly(ethylene oxide) fibers on M	Kanamycin	20	19
Centrifugation methods	PSA*	6.2	20
	Interleukin-6	65	21
Regulation of temperature and humidity	CRP	3	22
	Dengue virus DNA	10	22
Macromolecular crowders in running buffer	SARS-CoV-2, Strep-A	5-10	23
	Allergens	5-10	24
Laser-induced surface plasmon excitation	Cryptococcal antigen	32	25
Increase AuNP number			
Preassembled AuNP aggregates	HIV-1 nucleic acid	2.5	26
Enlargement of AuNP			
Catalytic reduction of Ag^+	Dengue virus protein	10	27
	Abrin-a	100	28
Decorating AuNPs with enzymes/nanozymes			
AuNP modification with HRP	DNA	10	29
AuNP functionalization with Pt	PSA	100	31

*AuNPs=Gold Nanoparticles; CP=Conjugate Pad; CRP=C-Reactive Protein; HepB=Hepatitis B; hIgG=Human Immunoglobulin; HRP=Horse Radish Peroxidase; LH=Luteinizing Hormone; M=Membrane; PSA=Prostate Specific Antigen; PVA=Polivinylalcohol; SP=Sample Pad; TZ=Test Zone.

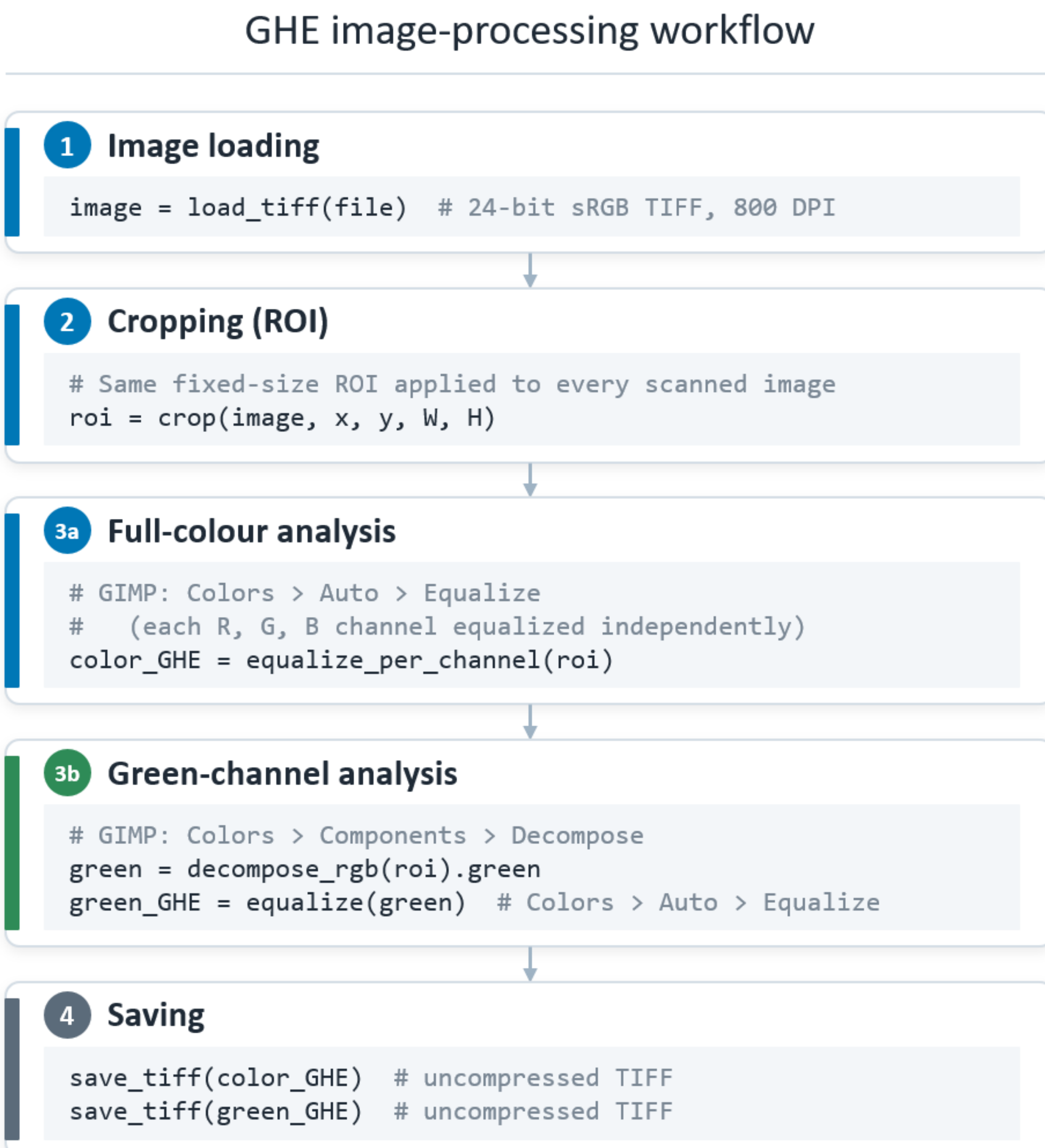

Figure S1

Figure S1.

Workflow in the forma of a pseudo-code, Python-style for the processing of lateral flow assay images by global histogram equalization (GHE). Functions shown are descriptive placeholders for the corresponding GIMP operations, not library functions.

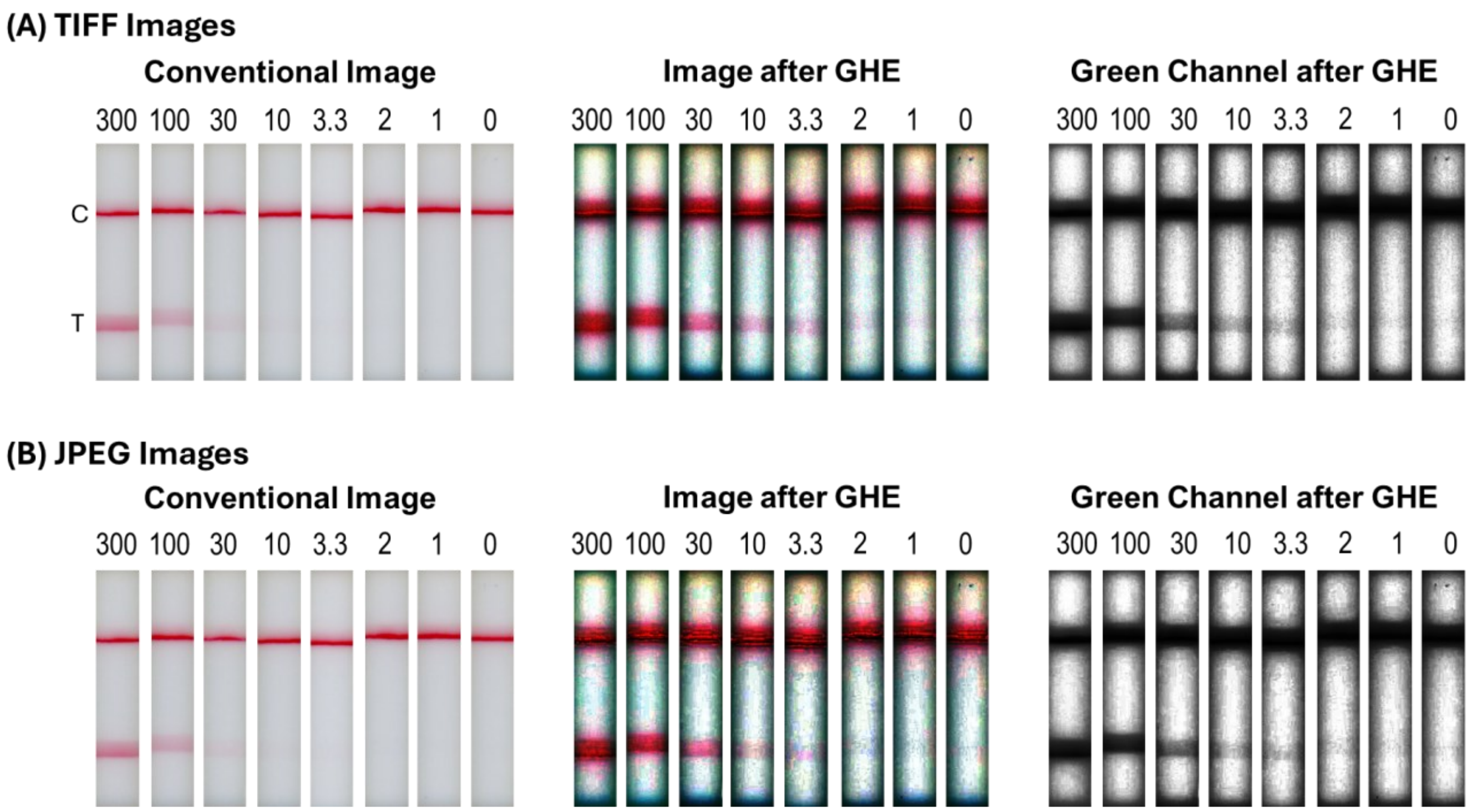

Figure S1

Figure S2.

Robustness of subvisual analytical signal recovery by global histogram equalization (GHE) across lossless and lossy image formats. **(A)** Lossless TIFF acquisitions. **(B)** Lossy JPEG captures. Both panels display conventional images alongside their post-GHE counterparts at nucleocapsid protein concentrations ranging from 0 to 300 pM (from left to right; 300, 100, 30, 10, 3.3, 2, 1 and 0 pM).
